# Pitfalls and Possibilities in the Analysis of Biomass Allocation Patterns in Plants

**DOI:** 10.3389/fpls.2012.00259

**Published:** 2012-12-05

**Authors:** Hendrik Poorter, Lawren Sack

**Affiliations:** ^1^Plant Sciences (IBG-2), Forschungszentrum JülichJülich, Germany; ^2^Department of Ecology and Evolutionary Biology, University of CaliforniaLos Angeles, CA, USA

**Keywords:** allometry, biomass allocation, leaf mass fraction, methodology, shoot:root ratio

## Abstract

Plants can differentially allocate biomass to leaves, stems, roots, and reproduction, and follow ontogenetic trajectories that interact with the prevailing climate. Various methodological tools exist to analyze the resulting allocation patterns, based either on the calculation of biomass ratios or fractions of different organs at a given point in time, or on a so-called allometric analysis of biomass data sampled across species or over an experimental growth period. We discuss the weak and strong points of each of these methods. Although both approaches have useful features, we suggest that often a plot of biomass fractions against total plant size, either across species or in the comparison of treatment effects, combines the best of both worlds.

## Introduction

How plants partition newly fixed carbohydrates among organs and biochemical fractions is likely to be as important to whole plant performance and ecology as photosynthesis itself. Carbohydrates may be employed to fuel leaf respiration, or can be stored as starch or fructans for later use. Alternatively, they can be transported elsewhere in the plant to be used to promote vegetative growth, maintenance processes, and/or reproduction. Integrated over time, the partitioning of carbohydrates to the various processes culminates in a plant with a given size and a so-called “biomass allocation” pattern. Detailed discussions on the terminology of allocation patterns and processes can be found in Litton et al. (2007) or Poorter et al. (2012). For the rest of this paper we will define “biomass allocation” as the realized distribution of biomass over the various organs of the plant, ignoring for practical reasons the carbohydrates and other compounds that disappeared from the plant during the processes of respiration, volatilization, or exudation.

As plants can invest most of their C only once, there are likely to be benefits and penalties under given sets of conditions for any specific distribution pattern of photosynthates to the various organs. The most effective biomass partitioning therefore depends on above- and below-ground resource availability. The investment pattern is thought to be optimal for resource foraging if all organs limit growth to the same extent (Bloom et al., 1985). Although species are constrained differently in their ranges of whole plant allocation patterns, they tend to show universal plastic responses, such as increasing mass allocation to leaf relative to stem plus root when growing in shade, but increasing allocation to root relative to leaf plus stem when growing in nutrient-poor soils (Poorter et al., 2012). Additionally, biomass allocation is strongly associated with growth form, and the niche they have evolved to occupy. For example, trees have much greater allocation to their stems relative to herbs (Niklas and Enquist, 2002). A rigorous analysis of biomass allocation patterns is crucial for evaluation of the performance of plants experiencing different environmental conditions, or in the comparison of growth of different species or genotypes within species.

Two classes of methods are in common use for analyzing biomass allocation patterns. The first class of methods, which we will denote as the “allocation approach,” employs biomass ratios, with the shoot:root ratio (S:R; Brouwer, 1963; Wilson, 1988) probably having been the most frequently presented variable. The S:R is intuitively appealing as a descriptor of the above- versus below-ground balance of biomass investments. However, other ratios are used as well, such as that for photosynthetic versus non-photosynthetic organs (Monsi and Saeki, 2005) or for leaf versus root (Kirschbaum et al., 1992). A related approach to describe allocation is by means of mass fractions, where the mass of the various organs is expressed relative to the total mass of the plant (Evans, 1972; Cornelissen et al., 1996; Poorter and Nagel, 2000).

The second class of methods is based on fitting so-called allometric equations. In its most typical form, the scaling among organs (i.e., relationship between the absolute size of one organ as a function of the size of another) is described by a power law with two parameters:

(1) $$Y=\alpha X^{\beta }$$

(Pearsall, 1927; Huxley, 1932; Niklas, 1994). This relationship can easily be converted into an equivalent linear version by applying a log-transformation to both sides of the equation (log *Y* = log α + β log *X*). If the equation is fitted to data for plants of a given species harvested over time, the parameter β has a clear biological and mathematical meaning, i.e., the ratio of the relative growth rates of the mass of organ *Y* relative to that of organ *X* (Huxley, 1932; Causton and Venus, 1981). The analysis is most straightforward when the plant is considered to consist of two components (e.g., above versus below-ground), but more complicated analyses are feasible. Huxley (1932) considered the log-transformed parameter α, the offset of the line at log(*X*) = 0, to be less interesting biologically, but it is nevertheless important for the interpretation as it indicates the relative scale of *X* and *Y* variables, given it equals the *Y* value at *X* = 1. Allometric analyses have been applied fruitfully to a range of botanical questions (Kohyama, 1987; Enquist and Niklas, 2002; Sack et al., 2003a,b; McCarthy and Enquist, 2007; Poorter et al., 2012).

With these two contrasting approaches available, it is important to know their weak and strong points for presenting and interpreting biomass allocation patterns. There have been several explicit statements in the literature pointing to the weak aspects of ratios, thereby promoting the allometric approach as superior (e.g., Jasienski and Bazzaz, 1999; Müller et al., 2000). In this paper we aim for a broad perspective, evaluating the pro and contra arguments for each approach, and suggest an analysis that combines the best of both methods.

## Materials and Methods

Evaluation of allocation patterns is often done for a given species treated with different levels of an environmental factor (e.g., Müller et al., 2000), or in the analysis of broad-scale patterns across a wide range of species (e.g., Niklas and Enquist, 2002). We compare the various analytical approaches using two datasets that are representative of these applications. The first dataset comes from an analysis of ontogenetic trends in biomass allocation for a given species, the bunch-grass *Deschampsia flexuosa*, when challenged with different levels of nutrients. Plants were cultivated in a climate-controlled growth chamber in large pots that were flushed each second day with a nutrient solution containing low or high-N, and harvested three times weekly. The substrate was coarse sand with a very narrow size distribution, which facilitated retrieval of virtually all roots. To allow comparison of plants over the same size trajectory, the high-N plants were sampled 16 times during 35 days, the low-N plants 22 times for 49 days. More details are given in Poorter et al. (1995).

The second dataset consists of leaf, stem, and root dry mass data for a diverse array of plant species of very different sizes and ages. This dataset consists of average values of harvests from a wide range of experiments, and, in the case of large trees, for plants harvested from plantations or other forest plots, with ∼5230 data entries for ∼250 herbaceous and ∼330 woody species. This database was previously used by Poorter et al. (2012) to compare allometric relationships across phylogenetic subgroups of the plant kingdom. All data were analyzed with the R statistical software (R Development Core Team, 2011).

## Results and Discussion

### 1 The allocation approach: Shoot-to-root ratio

**Figure 1:**
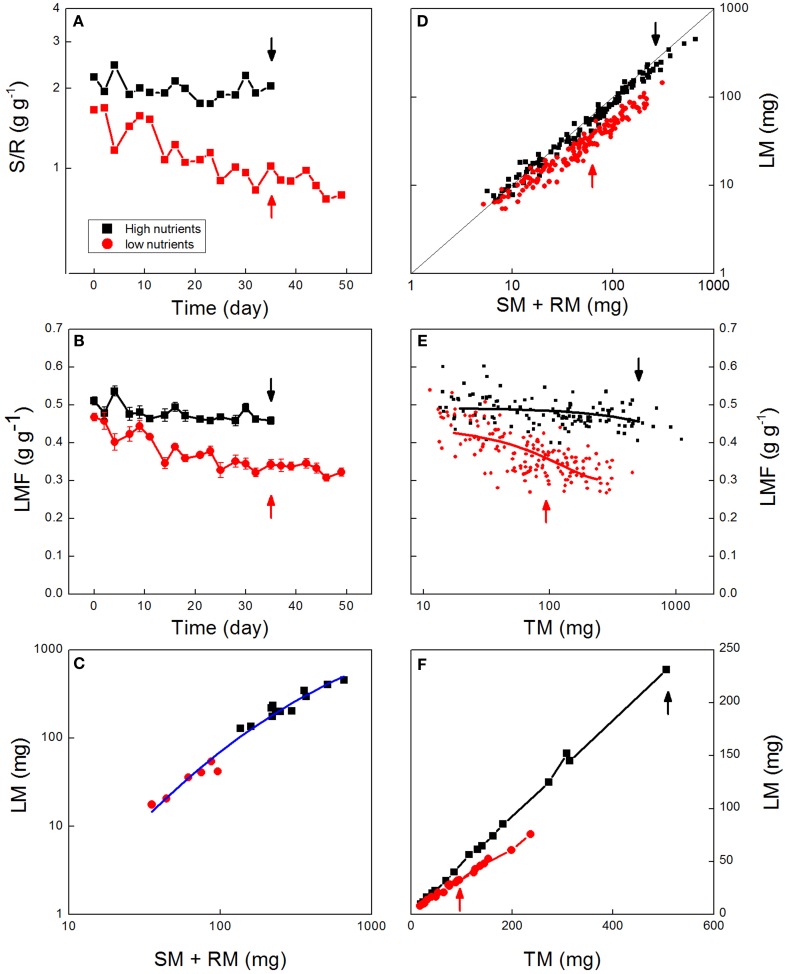
**Biomass allocation as affected by nutrient availability**. Data are shown from an experiment with *Deschampsia flexuosa* as described by Poorter et al. (1995). Data for high-nutrient plants are given in black, for low-nutrient plants in red. **(A)** Shoot:root ratio over time (Each point is the average of six sampled plants, except in the first and last harvest where *n* = 12). **(B)** Leaf Mass Fraction (LMF) values averaged per harvest and plotted against time. **(C)** Leaf mass (LM) plotted against stem plus root mass (SM + RM) of plants harvested at day 35. Points represent values for individual plants, and the line is the allometric equation fitted through all data. **(D)** Leaf mass plotted against stem plus root mass for all individual plants harvested throughout the experiment. The line indicates the 1:1 relationship. **(E)** Leaf mass plotted against total plant mass (TM) for low-nutrient and high-nutrient plants. Points represent values for all the individuals harvested during the experiment, and lines show the fitted polynomial trends. **(F)** Leaf mass plotted against total plant mass throughout the experiment. Points represent averages per harvest. Arrows indicate the plants harvested at day 35. All biomass values are based on dried material.

Ratios are often used in the literature to standardize biological data. They are flexible in that they can be applied to two variables, even when the variables have disparate units (Liermann et al., 2004) and have particular appeal when they encapsulate data to clearly reflect biological concepts. As described above, a popular biomass allocation ratio is the S:R ratio, which summarizes the trade-off between above- and below-ground investments. In case of the experiment with *Deschampsia flexuosa* the S:R is rather stable over time in the high-nutrient plants, but steadily decreases in the nutrient-stressed plants (Figure 1A), consistent with ongoing plastic adjustment to increase below-ground resource capture.

Possible pitfalls, disadvantages, and/or points requiring attention in the application of ratios such as S:R fall into two categories. First, there are some statistical issues:

#### A Unboundedness, asymmetry, and non-normality of ratio values

By nature, ratios are non-normally distributed (Sokal and Rohlf, 1995). If a plant has an equal mass in two organs A and B, then A:B = 1. If the mass of organ A is larger than that of organ B, the ratio can in principle range to infinity; however, if it is organ B that is larger, the ratio can only decrease to 0. Log-transformations are then required to make the data amenable to statistical analyses, including very basic calculations such as averages. As an example, assume that plant *x* has a shoot mass of 2 g and a root mass of 1 g, whereas plant *y* has, just by chance, the opposite allocation pattern. The S:R values are then 2 and 0.5 for plants *x* and *y* respectively, which gives an average of 1.25. A log_2_ transformation on the two plants’ S:R values results in values of 1 and −1, with 0 as an average, and a back-transformed S:R of 1, which is the appropriate estimate of the average in this case.

#### B Difficulty of comparing different ratios, especially at low values

Ratios such as S:R with values less than 1 are less easy to visualize and work with, and thus, depending on species and conditions, some studies present R:S rather than S:R values. Although both are equally valid in representing data, alternative use of these inversely related expressions becomes confusing when one tries to make generalizations across a body of literature, as a seemingly large increase in S:R from 2 to 3 is exactly equivalent to a R:S decrease from 0.50 to 0.33.

#### C Ratios are characterized by a relatively high variability

Variability in both the numerator and the denominator contribute to the total variance of a ratio (Sokal and Rohlf, 1995). There is no simple formula to estimate the variation in the ratio based on the variance of its components (see Holmes and Buhr, 2007 for approximations). This phenomenon of inflated error in the ratio can reduce power when testing for differences among treatments in calculated ratios (Jasienski and Bazzaz, 1999). It is especially problematic when numerator and denominator values vary completely independently of each other. In the case of correlated variables, the effect is smaller. Leaf, stem, and root mass data for individual plants are generally highly correlated, especially when considered after log-transformation (Table 1).

**Table 1:** **Correlation coefficients among leaf (LM), stem (SM), root (RM), and total plant mass (TM), both given before (black) and after (blue) log-transformation**.

	LM	SM	RM	TM
**A**				
LM	–	**0.98**	**0.98**	**1.00**
SM	**0.99**	–	**0.98**	**0.99**
RM	**0.99**	**0.99**	–	**0.99**
TM	**1.00**	**1.00**	**0.99**	–
**B**				
LM	–	0.94	**0.96**	**0.99**
SM	**0.97**	–	0.93	**0.95**
RM	**0.97**	**0.95**	–	**0.99**
TM	**0.99**	**0.97**	**0.99**	–
**C**				
LM	–	0.68	0.71	0.70
SM	**0.99**	–	0.93	**1.00**
RM	**0.99**	**0.99**	–	**0.96**
TM	**0.99**	**1.00**	**1.00**	–

*A. *Deschampsia flexuosa*, grown at a high-nutrient level (*n* = 119). B. Idem for plants grown at low-nutrient supply (*n* = 155) C. For a wide compilation of biomass data from the literature (*n* = 5230). Values above 0.950 are given in bold. All biomass values were for dried material*.

The second group of issues relate principally to the biological interpretation:

#### D Loss of biological information when using ratios

Ratios can encapsulate only the two quantities from which they are calculated. Most plants have distinct leaf, stem, and root organs contrasting in their functions. When relying on one ratio, two of the organs have to be combined (such as leaves and stems in the S:R ratio, or one organ has to be neglected (such as in the leaf:root ratio). In all cases, the use of one ratio entails a loss of information about the actual allocation pattern in the plant. For example, Poorter et al. (2012) found that plants grown at high densities allocated more strongly to stems than plants grown at low densities, at the expense of investment in both leaves and roots. However, this pattern was not obvious from the S:R data.

#### E Difficulty in interpreting differences in fractions without deeper investigation

Ratios can be affected in various ways, as a shift in the ratio can be due to a change in the numerator or the denominator, or both. In the case of the experiment with *Deschampsia*, for example, where low-nutrient plants develop lower S:R values than control plants (Figure 1A), it is not clear whether this lower S:R arose from slower growth of the leaf fraction, a faster growth of roots, or a combination of both.

#### F Differences in biomass allocation ratios can confound ontogenetic effects with treatment and species differences

Ratios can be potentially misleading when plants of different sizes are compared (Packard and Boardman, 1988; Jasienski and Bazzaz, 1999; Müller et al., 2000; Weiner et al., 2009). If the relationship between the masses of the various organs is linear and goes though the origin, it is called “isometric.” This special condition does not impose any interpretation problem; the ratio is preserved across the range of organ sizes. However, if the relationship is curved, or does not intersect the origin, the relationship is called “allometric,” and this means that the ratio changes with the size of the plant. Suppose that larger plants generally have higher S:R, and plants grown with a high-nutrient supply are larger *and* have a higher S:R than low-nutrient grown plants (Figure 1A). In that case it is difficult to say whether the higher S:R value of the high-nutrient plants was simply the expression of baseline development of form during ontogeny, active reprogramming of the plant to acquire more of the limiting resource (light rather than nutrients in this case), or both (Coleman et al., 1994). If the aim is to show that plants actively reprogram their allocation pattern for any specific environmental challenge, analysis of biomass ratios at a given moment of time are not sufficient to tease these two possible contributing processes apart.

### 2 The allocation approach: Biomass fractions

Some of the problems mentioned above can be avoided by using fractions or percentages rather than unbounded ratios. Fractions and percentages are omnipresent in many facets of social, financial, and political life, for good reason as they provide a very easily interpretable variable. The reason for that is that the component values always add up to 1.0 or 100, thereby providing an easy-to-understand scaling. In any framework based on the C-economy of plants, fractions are easy to apply and come close to completely summarizing how plants partition available photosynthates over the various organs. The use of fractions avoids some of the complications related to ratios. Specifically, fractions are not confined to representing only two of many components (see point 1D). Generally, three fractions are calculated for a vegetative plant: LMF, SMF, and RMF, representing the leaf, stem, and root mass fraction, respectively. The number of fractions can be expanded, however, if one would like to separate the fine root fraction from the other roots (Körner, 1994), for example, or consider reproductive organs as well. Biomass fractions can highlight changes in allocation over time and how these vary with given conditions. In the case of *Deschampsia flexuosa*, the LMF slightly decreased over time for the high-nutrient grown plants, whereas the decrease was much stronger in the low-nutrient grown plants (Figure 1B). At the final harvest, LMF was 0.46 for the high-nutrient plants, and 0.31 for the low-nutrient plants, representing a substantial and statistically highly significant (*P* < 0.001) difference in allocation to leaves.

Statistical pitfalls, disadvantages, and/or points requiring attention in the application of fractions include:

#### A Non-normality of fraction values

Fractions are not normally distributed, although the distribution is far less skewed than in the case of ratios (see point 1A). The arcsin transformation is a popular transformation (Sokal and Rohlf, 1995), but others, like the logit-transformation can be useful as well (Warton and Hui, 2011). Practically, these transformations do not necessarily lead to very different conclusions, and the effect of the transformations is often negligible. This is especially the case if the various fractions of the plant have values between 0.20 and 0.80. Adult trees exempted, most LMF and RMF values are within that range (see Poorter et al., 2012).

#### B Non-independence of numerator and denominator

Biomass fractions have also been criticized, as the mass of the organ of interest influences both the numerator and denominator. Hence, numerator and denominator are not fully independent (Müller et al., 2000). The concern with this situation is possibly rooted in the classical paper of Pearson (1897) on “spurious” correlations derived from ratios of independent variables. Pearson analyzed what happens if two unrelated variables A and B are divided by a third unrelated variable C. The resulting two ratios will show a positive correlation, even though the underlying variables were all in fact unrelated. Thus, scientists have to be aware of the possibility of spurious relationships when they correlate various ratios (Weller, 1987) and adjust their null model when such correlations are tested (Jackson and Somers, 1991; Brett, 2004). However, in our view this mathematical tendency toward autocorrelation of ratios does not invalidate the use of fractions or percentages *per se* as simple descriptors of how parts of an organism relate to the whole plant, and real biological implications can be inferred from these variables.

Pitfalls and shortcomings of fractions related to the biological interpretation are partly overlapping with those mentioned for ratios.

#### C Difficulty in interpreting differences in fractions without deeper investigation

It is unclear whether changes in fractions are more strongly influenced by changes in the numerator, the denominator, or both (see point 1E).

#### D Differences in biomass allocation fractions can confound ontogenetic effects with treatment and species differences

Fractions can be misleading for considering changes in biomass allocation in growth treatments, as this approach cannot tease apart baseline developmental changes during ontogeny from active reprogramming of biomass allocation in given treatments (see point 1F).

### 3 Allometry

The main alternative to ratios and fractions is the analysis of allocation within an allometric framework. Time *per se* is not an explicit parameter in this analysis, as log-transformed sizes of two organs at given points in time are plotted against one another. Allometric analyses are ideal to analyze which of the organs changes its growth response relative to the others, and at what size this occurs. Thus, the allometric analysis is specifically able to circumvent the problems mentioned in 1F and 2D. Further, because this analysis does not include an additional calculation, it is not affected by the variability of having a numerator and denominator, a downside of using ratios described in section 1E.

Statistical pitfalls, disadvantages and/or points requiring attention in the application of allometry include:

#### A Misapplication of log-linear model

**Figure 2:**
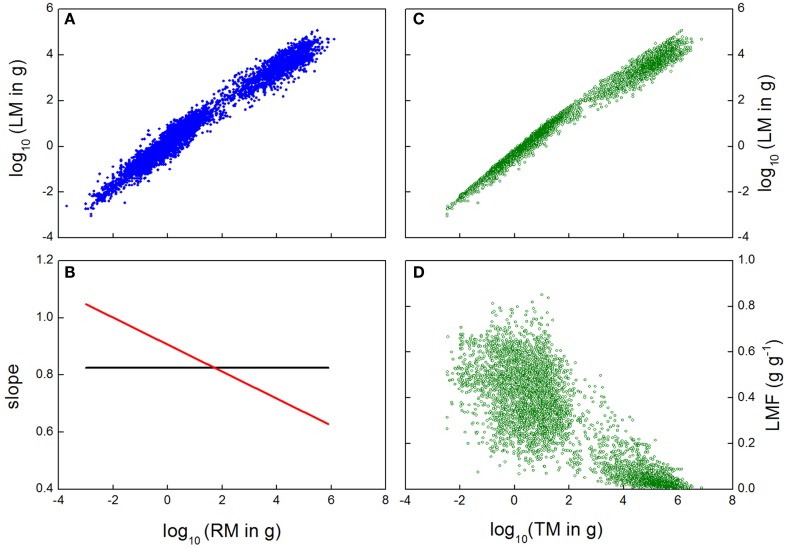
**(A)** Log-transformed leaf mass (LM) plotted against log-transformed root mass (RM) for a wide range of species and growth conditions. **(B)** Slope of a linear (black) or quadratic (red) relationship fitted through the data of **(A)**. **(C)** Log-transformed leaf mass data plotted against log-transformed total plant mass (TM), and **(D)** Leaf Mass Fraction (LMF) plotted against log-transformed total plant mass for the same observations. Data are from Poorter et al. (2012), and comprise a large dataset (*n* = ∼5230) of leaves, stems, and roots of herbaceous and woody species.

As mentioned above, the analysis is typically carried out by fitting a straight line through the log-transformed data for organ mass values. Although plots of mass values of plant components may often be linear on a log–log scale, or at least appears so, careful consideration may reveal this is not always true (Causton and Venus, 1981; Bernacchi et al., 2000). In the case of our across species data set, a straight line fitted to log–log data could explain 97.2% of the variation in size of the leaves versus roots (Figure 2A), with a quadratic equation improving the fit to 97.5%. While the first value seems highly impressive, and the improvement in fit by the quadratic term only marginal, even minor deviations from the log–log line imply large changes in allocation patterns. Automatic application of a linear log–log relationship can miss out on this variation, with potentially serious problems for testing theory. West et al. (1999), for example, attach a biological meaning to the exact value for the slope of the linear log–log relationship, but such interpretation would be inappropriate if the log–log relationship turns out not to be linear at all. Indeed, in our data the slope was ∼0.82 with the linear fit, but decreased from 1.05 to 0.63 for the quadratic fit (Figure 2B). Therefore, careful selection of the appropriate model is of prime importance in the allometric analysis.

#### B Choice of line-fitting method can affect results

Beyond the choice of the structural model (i.e., linear versus quadratic fit to the log–log data), different statistical tests for fitting that model to data may give different estimates of parameters such as the slope of the line (Niklas, 1994; Müller et al., 2000). Allometry studies typically use standard major axes (SMA) or ordinary linear regression (OLS) for fitting the line to log–log data. The case has been made that the SMA is always most appropriate for allometric scaling analyses, because it assumes no dependent variable, and accounts for measurement error and natural variation in both variables; by contrast, the OLS accounts for error only in the *y*-variable and not the *x*-variable and is thus appropriate only for prediction (Warton et al., 2006). However, simulation studies have shown that when measurement error or random variation in the *y*-variable is substantially larger than that in *x*, the SMA is a biased estimator, and the OLS provides more accurate results (McArdle, 1988; Kimura, 1992). Notably, when the *r*^2^ of the relationship is very high, either line-fitting method will produce similar parameters (McArdle, 1988; Niklas, 1994).

A related, but little-discussed problem with the SMA-derived parameters may show up when various fitted allometries are combined algebraically, e.g., if leaf area is estimated from stem diameter based on algebraically determining a leaf area-stem diameter relationship from fitted allometries for leaf area versus plant height, and for plant height versus stem diameter. This estimation can be poor if based on SMA-derived allometric parameters, especially at lower *r*^2^. The algebraically derived allometric slope will then deviate from the data (see Table S1 in Supplementary Material). Thus, it is necessary to use OLS for fitting allometries when the errors in the two variables differ strongly, or if the allometries are to be combined algebraically to derive another allometry.

#### C Risk of (partial) extrapolation of data trends

Partial or complete extrapolation of fitted trends can result in misleading analyses of treatment effects. As an example, Müller et al. (2000) investigated plant responses to two levels of nutrient supply for 27 species. Shoots and roots were harvested for 9–11 individuals of each species in each treatment at the end of the growing season. For most species Müller et al. (2000) observed a significant difference in S:R between high- and low-nutrient plants. However, using the allometric analysis for each species, the plants in both treatments followed the same relationship. The authors therefore concluded that the low-nutrient plants did not actively adjust their allocation, but that the variation among treatments was simply an effect of shared ontogenetic trajectories, with differences in S:R arising simply from the larger plant sizes in the high-nutrient treatment. However, such a conclusion could be misleading if based only on that analysis. Using the data for *Deschampsia flexuosa* we found the same result when we plotted data for the last day that plants were harvested simultaneously for both treatments (day 35). That is, the low-nutrient plants had a significantly lower LMF and a higher RMF (*P* < 0.001), but in an allometric plot all data points apparently fell around the same curve (Figure 1C). Notably, both in the analysis of Müller et al. (2000) and in ours in Figure 1C, plants of the two treatments did not overlap in their sizes, and the comparisons of the allometries in the two treatments in this analysis were based fully on extrapolation. Is such an extrapolation justified? To truly confirm this finding of a common allometry across nutrient treatments, it is necessary to consider plants over a range of harvests which overlap in sizes. When we conducted that analysis for our full dataset, it was clear that plants from the two nutrient treatments followed different trajectories (Figure 1D), distinct both in allometric slopes and *y*-intercepts. Thus, in contrast to what would be concluded from an allometric analysis of final harvest data, allometric analysis of *all* the data showed a substantially lower LMF for the low-nutrient plants whether considered for plants of the same age, or of the same size (*P* < 0.001; see the arrows in Figure 1), supporting the conclusion that true shifts in the program of biomass allocation occurred between treatments. Therefore, in allometric analysis care should be taken not to rely on data obtained from one-harvest only, but spread harvesting over the full growth period (Coleman et al., 1994), such that allometries can be analyzed for the same range of mass values, avoiding extrapolation.

#### D Power of the statistical test

In many cases in the literature, the conclusion of comparison of allometries for plants of given species grown in different treatments is that the differences in biomass allocation which were observed as S:R or LMF at a common point in time disappeared after allometric correction (e.g., Gunn et al., 1999). One would expect that there would be a similar number of occasions where the reverse is observed, with no significant difference in the biomass allocation pattern at a common time point but a clear difference after size correction. Intriguingly, such observations are hardly ever reported in the literature. This could be a true biological phenomenon, something that is simply never tested, or it has not been considered relevant by the authors to discuss in their papers. Alternatively, it may also be caused by the statistical analysis related to the allometric test (comparison of two slopes with 1 degree of freedom) being less powerful than the statistics used to test differences in mass fractions (comparing averages of individual plant observations with many degrees of freedom). Such a case has been demonstrated by Poorter and Nagel (2000), but has to our knowledge never been systematically explored.

Issues related to the biological interpretation are the following:

#### E Loss of information of discrete, distinct growth stages

Generally, the growth of different organs is very well coordinated (Brouwer, 1963). However, there are cases reported where priority of plant growth shifts discretely with ontogeny, with growth focused more on roots or shoots for specific periods of the year (Drew and Ledig, 1980; Danner and Knapp, 2001). Consequently, LMF and RMF may show distinct cycles of increases and decreases. This kind of dynamic will not be visible from the allometry of log shoot versus log root, except as noisy points around the mass of the plant when the growth shifts.

#### F Difficulty in inferring or proving a specific biological meaning for allometric parameter values

As discussed above, the allometric slope β for log *Y* versus log *X* is equal to the ratio of the RGR’s of *Y* and *X* (where, e.g., *Y* and *X* could be, e.g., shoot and root mass). Thus, the slope β can be taken as a biologically meaningful quantity when comparing plants of a given species during ontogeny, and possibly for closely related species within a genus, to represent trends during evolution (Gould, 1966). However, β can be unity with LMF being very low or very high, depending on the value of log(α). Similarly, plants of given species grown in different treatments, or plants of different species could have exactly the same LMF with very different combinations of β and log(α). Therefore, as both β and log(α) are required to interpret the actual allocation, these parameters do not convey the allocation pattern as transparently as fractions.

#### G Underestimation of the biological variation in allocation based on the fit of the allometry

It is very important to understand that a fitted allometry represents a central trend, and for log–log allometries, a high *r*^2^ can simply be caused by a wide range of size values, For example, McCarthy et al. (2007) presented data for a wide range of species and biomass, with almost eight orders of magnitude of variation in plant mass. They fitted a linear equation through the log-transformed data, and concluded that “plant size explained 97–99% of the variation in biomass distribution to leaf, stem, and root”. Notably, the idea that the *r*^2^ of the allometric relation represents the variation in biomass *allocation* is not accurate. The *r*^2^ of 0.97 indicates that on this scale with an 8-fold order of variation in magnitude, one is very well able to predict the average shoot mass if one knows the average root or total plant mass. In other words: plants with large leaf mass are very likely to have large stem and root mass, and plants with small leaf mass will also have small stem and root mass. However, the *r*^2^ value does not necessarily indicate the precision of the actual biomass *allocation*, as there is still large variation that is orthogonal to plant mass, which is obscured by the log–log plot which collapses the spread of the points. The actual variation in biomass allocation across plants of given sizes in our across species dataset, even given the *r*^2^ of 0.99 in the allometric plot of leaf mass versus plant size (Figure 2C), is clarified when the same biomass data are used to calculate the LMF of the plants, and plot these against total plant size (Figure 2D). This graph gives a very different perspective, as the variation in allocation now is more visible, with an *r*^2^ of 0.71. The risk of misinterpretation of high correlations over large ranges of values is not limited to plant or animal sizes only. In the analysis of genome-wide correlations of RNA expression among treatments or genotypes, any random comparison will show correlation coefficients of 0.80 or higher (Giorgi et al., 2010), simply because some genes will always have high expression and others low.

#### H Lack of quantitative estimates

Another point of concern is that most allometric analyses stop at the point of statistical analysis showing the *presence* of a size-independent effect of a given treatment (e.g., Harmens et al., 2000). However, the actual *magnitude* of the treatment effect, after correction for possible differences in plant size, is still not known. We need this information for further quantitative analysis, such as a meta-analysis of size-corrected allocation values. In the case of the nutrient experiment, there was indeed a significant difference in β, with the value at the high-nutrient level being higher than for the low-nutrient plants (Table 2). The difference in log(α) was not statistically significant. However, when it comes to the actual allocation pattern, it seems that the direct test of LMF differences is more informative. Over all three size classes considered (10–30, 30–100, and 100–300 mg), the high-nutrient plants had almost 50% of their biomass invested in leaves, whereas the low-nutrient plants showed a steady decrease in LMF with size, going down to an average 33% biomass invested in leaves (Table 2). In all size classes the LMF difference was statistically highly significant.

**Table 2:** **Comparison of the output of analysis of LMF and allometry for the *Deschamspia flexuosa* plants grown at a low or high-nutrient supply, and the number of plants in the respective analysis**.

	Low-N	High-N	*P*
LMF (plants in 10–30 mg range)	0.444	0.504	***
No. of plants in 10–30 mg range	30	20	
LMF (plants in 30–100 mg range)	0.369	0.491	***
No. of plants in 30–100 mg range	73	31	
LMF (plants in 100–300 mg range)	0.333	0.472	***
No. of plants in 100–300 mg range	48	48	
Log(α)	0.077	0.059	ns
β	0.811	0.945	*

Significance values: *: *P* < 0.05; ***: *P* < 0.001. Note that the R module used to test the difference in β in the standard major axis analysis (lmodel2) only provides 95% confidence intervals.

#### I Lack of clarity in presentation

If one were interested only in a correction for plant size for biomass allocation, presentation of the results is straight forward. But what if one would like to correct a range of very different variables for ontogeny? Following the same allometric methodology, for the analysis of leaf N concentration, one would plot total leaf N versus total leaf mass, for root C it would be total root C against root mass, and for photosynthesis it would be total carbon fixation against total leaf area. For other variables, such as δ^13^C values, there is no obvious counter variable of choice. Presenting this variety of plots, all with different *x*-axes, hampers a simple and consistent overall analysis of the data.

### 4 Analysis of fractions versus size: Some of the advantages of allometry with additional benefits

Having discussed the potential pitfalls of the two main types of analysis, we next focus on two additional approaches that incorporate aspects of both biomass allocation fractions and allometry. As mentioned above, the allometric analysis offers the unique advantage that the exact relationship between two compartments can be analyzed, without interference from changes in other compartments. Thus, the allometric relationships provide clarity on the developmental coordination of the growth of different organs, and how this coordination is affected by treatments, or how it varies across species. However, in most ecophysiological research, it is not only the coordination of growth processes that is important, but also the actual values of LMF, SMF, and RMF *per se*, because these are important variables in the models of growth and equations that determine the uptake of carbon, nutrients, and water (Evans, 1972; Garnier, 1991; Poorter, 2002). For this reason, we advocate an analysis where the treated data remain close to the biological parameter of interest (Prairie and Bird, 1989), unless there are compelling reasons to do otherwise. A simple plot that provides clear detail on the ontogenetic shift with plant size is that of LMF (or any other parameter of interest) plotted directly against total plant mass, for all treatments and over the full range of harvests. This approach has been used by, e.g., Evans (1972); Poorter and Pothmann (1992); Walters et al. (1993) and Xie et al. (2012). It allows for a straight comparison of the parameter of interest, i.e., LMF, over the full range of total plant masses studied. Using the data for *Deschampsia flexuosa* this approach revealed a robust and significant difference (*P* < 0.001) in LMF between the high and low-N treatments over almost the full size trajectory (Figure 1E). The results of this analysis are very similar to those given by the allometric analysis (Figure 1D). Both analyses show a clear shift in allocation across treatments for plants of equal size. However, in Figure 1E we not only visualize a systematic difference in allocation, but can also determine a quantitative estimate of its magnitude (see section 3H).

Plotting LMF directly against log-transformed total plant mass, as shown in Figure 1E avoid several other problems of the allometric approach. One advantage is that statistical tests can be confined to those data points that are present for the same size range, avoiding the problem of extrapolation described in section 3C. Further, we can see shifting phases in growth – e.g., if the plant maintains a high RMF during early growth, and then increases in LMF thereafter. Such trends will not be picked up in allometric plots (see point 3E). Finally, this analysis addresses the problem mentioned in section 3I as all variables are corrected for size in the same way and using the same parameter (total plant mass). Further, this type of analysis also avoids the problem of the allocation approach, where two ratios could be equal with very different values in the two numerators and denominators (point 1E and 2C). As fractions are now plotted in combination with total plant mass, there is no degree of freedom left anymore for the component values, making the characterization of allocation at any plant size fully defined.

We recommend that for studies emphasizing biomass allocation (and not the coordination of the growth of organs, *per se*) both the allometric analysis and plot of fractions versus size should be used for maximum benefit. If differences are observed in the results of the analysis, then we suggest investigating the reason, and presenting the plot of biomass fraction versus plant size as most directly relevant.

### 5 Partitioning coefficients: High resolution detail of allocation

**Figure 3:**
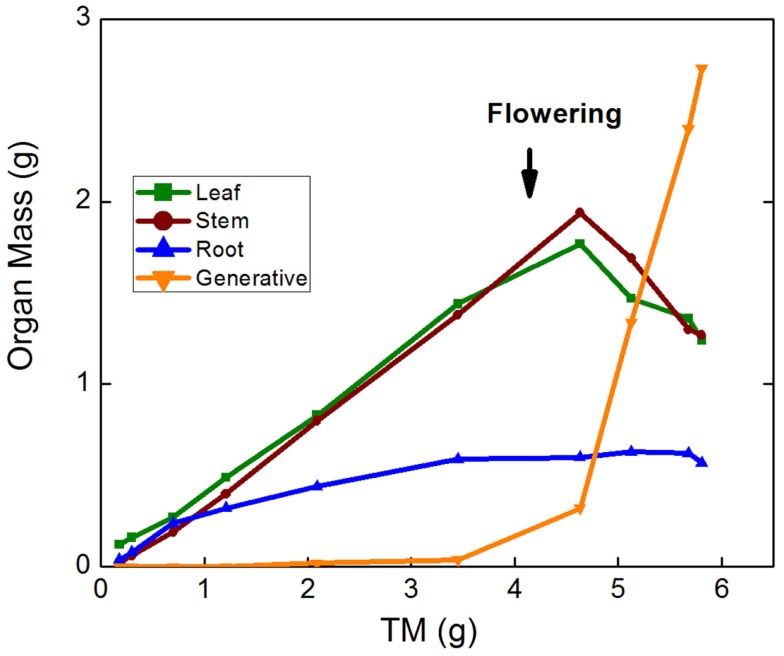
**Leaf, stem, root, and reproductive mass of *Pisum sativum* plotted against total plant mass (TM)**. Plants were grown hydroponically in a growth room from the seedling to the generative stage (P. W. Wolswinkel and T. L. Pons, pers. comm.).

A further approach to analyzing allocation over time is a graphical analysis of how the mass of a particular organ changes when plotted against total plant mass (Huxley, 1932; van de Sande-Bakhuyzen, 1937). This is a hybrid of the analyses of Figures 1D and 1E. The slope of these plots can be considered as a “partitioning coefficient,” which truly indicates the proportion of newly formed biomass invested in the various organs at any given moment in time. Partitioning coefficients are generally constant over prolonged phases in the development of the plant (Trapani et al., 1994). In the case of the *Deschampsia* data, small differences in slope occurred during the first part of the experiment, but generally the slopes remained rather stable over time (Figure 1F), with partitioning coefficients to leaves of 46 and 30% for high-nutrient and low-nutrient plants, respectively. A similar analysis for *Pisum sativum*, including the generative phase, showed marked changes in allocation, where root growth and subsequently leaf and stem growth ceased soon after flowering, followed by strong biomass redistribution from stems and leaves to the fruits, but not from roots (Figure 3).

### 6 What is the “best” approach?

We described several ways to analyze biomass allocation, and their strengths and weaknesses. However, it is not possible to conclude which of these methods is best, as each has strengths and pitfalls, and the pertinent analysis will also depend on the specific question asked. Even in the case of one-harvest experiments, it is relevant to determine the biomass fractions, as such information is essential to understand the C-budget of such plants, of which LMF is a component. If information is desired on the impacts of treatments or the differences among species, independent from variation in plant size, then an analysis that corrects for size is necessary. This requires multiple harvest data, where analyses of graphs of biomass fractions over time and against plant size, as well as allometric plots will provide important insights. Of these three, we suggest that the plots of biomass fractions against plant size often provide the most direct and instructive information. Allometric plots may provide additional information on the coordination of growth processes, with both fractions and allometry subject to the assumptions and pitfalls described above.

## Conflict of Interest Statement

The authors declare that the research was conducted in the absence of any commercial or financial relationships that could be construed as a potential conflict of interest.

## Supplementary Material

The Supplementary Material for this article can be found online at http://www.frontiersin.org/Functional_Plant_Ecology/10.3389/fpls.2012.00259/abstract

Supplementary Table S1 **Allometric Algebra**. The effect of OLS and SMA fitting in the case where allometries are algebraically combined.
